# Children’s Use of Race in Their Social Judgments: A Multi-Site, Multi-Racial Group Comparison

**DOI:** 10.1525/collabra.132489

**Published:** 2025-04-01

**Authors:** Mercedes A. Muñoz, Elizabeth A. Enright, Sarah E. Gaither, May Ling D. Halim, Kristin Pauker, Kristina R. Olson, Yarrow Dunham

**Affiliations:** 1 Psychology & Neuroscience, Duke University, Durham, NC, USA; 2 Psychology, St. Mary’s College of Maryland, St. Marys City, MD, USA; 3 Psychology, California State University, Long Beach, CA, USA; 4 Psychology, University of Hawaii at Manoa, Honolulu, HI, USA; 5 Psychology, Princeton University, Princeton, NJ, USA; 6 Psychology, Yale University, New Haven, CT, USA

**Keywords:** intergroup processes, social judgements, race

## Abstract

Studies assessing children’s use of race in social judgment have often focused on White participants and usually include targets of only one or two racial backgrounds. They have also employed a wide range of methods, making comparisons across studies difficult. In this paper, we recruited a large sample of children ages 4- to 6-years-old (*N* = 666) belonging to the United States’ four largest racial/ethnic groups (Black, Latine, Asian, and White) in five geographic regions (Durham, NC; Honolulu, HI; Long Beach, CA; New Haven, CT; Seattle, WA) to broadly examine children’s race-based social judgments (including measures of racial attitudes, interpersonal distance, resource allocation, and status perception). Overall, children demonstrated consistent ingroup biases in the attitudes, resource allocation, and interpersonal distance measures, but did not systematically associate their ingroup with higher status. When analyzed separately by participant race, White children tended to show these effects at above chance rates, sometimes significantly more than children in other racial groups. Results for Black, Latine, and Asian children were more variable across measures.

## Introduction

The United States is becoming increasingly racially and ethnically diverse, as demonstrated by changes to the reported demographics of its public school students. In 2000, approximately 61% of students enrolled in public elementary and secondary schools identified as White and 37% identified with a racial/ethnic minority group. In 2021, 45% of public school students identified as White and 55% of students identified as a racial/ethnic minority (De Brey et al., 2023). With this demographic shift comes more opportunities for children to interact with peers from different racial backgrounds, leading parents, educators, and researchers alike to inquire about how or when children in the United States (U.S.) are considering race in their social judgments. Having a better understanding of racially diverse U.S. children’s race-based social judgements and when they develop can be generally beneficial to understanding children’s interpersonal relationships. Additionally, it may allow us to identify potential sensitive periods for racial bias reduction, which could lead to the development of interventions to modify observed biases that could otherwise lead to more harmful racial prejudice in adulthood.

Existing research suggests that by the preschool years children will use race and other ingroup characteristics to inform their social judgments. Throughout this paper, we use the term *social judgments* to refer to a set of measures that include assessments of social attitudes (measures that assess good/bad judgments or liking evaluations of racial groups), resource allocation (measures that assess giving behaviors towards different racial groups), interpersonal distance (measures of physical closeness or distance from other racial groups), and status (measures assessing perceptions of social hierarchy, power, wealth and/or dominance of racial groups). For example, 5-year-old children say they would rather be friends with a same-race peer (Shutts et al., 2011), 3–11-year-old children allocate more resources to others who share their race (Renno & Shutts, 2015; Zinser et al., 1981), 5-year-old children prefer to be physically closer to children of their own gender (Halim et al., 2017), and 3- to 11-year old children say a White adult is “in charge” when compared to a Black adult (Dukler & Liberman, 2022).

Despite these general findings, not all studies or all samples of children show these effects, nor to the same degree (e.g., Elenbaas & Killen, 2016; Kinzler & Spelke, 2011; Mandalaywala et al., 2021; Marshall et al., 2022). For example, while most research has found that White children show consistent pro-White biases (Aboud, 1993; Newheiser & Olson, 2012; Zinser et al., 1981), research with racial/ethnic minority children has found more variability, with some showing pro-White biases and others showing ingroup preferences (Brand et al., 1974; Elenbaas & Killen, 2016; Jordan & Hernandez-Reif, 2009; Kircher & Furby, 1971; Marshall et al., 2022). Previously, variation in the strength of race-based bias judgments or lack thereof has been attributed to differences across age (e.g., Cooley & Killen, 2015; Elenbaas & Killen, 2016) and environmental differences (e.g., Setoh et al., 2023; Shutts et al., 2011). Direct comparisons are difficult, however, because different studies adopt different methods, scoring, and stimuli. Further, participants across studies also often vary across ages, racial backgrounds, and environmental contexts.

In order to better understand preschool children’s contemporary racial judgments, the current paper recruited 4- to 6-year-old children from the United States’ four largest racial/ethnic groups (henceforth *racial* groups for brevity): White, Hispanic/Latine (henceforth *Latine* for brevity), Black, and Asian children. These participants were recruited from across five distinct geographic locations throughout the United States (Durham, NC; Honolulu, HI; Long Beach, CA; New Haven, CT; Seattle, WA), but all were given the same battery of measures administered in the same way. By standardizing the administration of racial bias measures across geographic regions and varying participant races, we are better able to compare the race-based social judgments of a diverse set of children. Additionally, we are able to explore the prevalence of ingroup racial bias in both White children and minority children.

### Preschool Children’s Race-Based Social Judgments

People are thought to prefer their ingroup for a variety of different reasons, including to promote ingroup cooperation, harmony, and positivity (Brewer, 2007; Sumner, 1906). Social Identity Theory posits that ingroup preferences emerge from a motivation to enhance one’s self-esteem in a search for positive social distinctiveness (Nesdale & Flesser, 2001; Turner et al., 1979). This tendency to favor one’s ingroup appears to emerge early in development, as even children as young as 3 will favor a novel or arbitrary group they are assigned to (Richter et al., 2016).

Children’s ingroup judgments are often assessed in the domain of race, because race is highly salient in the contemporary U.S. By the preschool years, young children use race when evaluating others (Waxman, 2021). In one of the earliest studies of children’s racial judgments, Clark and Clark (1947) asked 3- to 7-year-old Black and White children to choose whether a Black or a White doll was “nice”, “good”, and whether it had other positive and negative attributes. Ultimately both White and Black children assigned more positive attributes to the White doll. Much of the existing literature on children’s racial judgments has focused on White children, who are the numerical majority group in most of the countries where this type of research has been routinely conducted (e.g., Australia, Canada, the United Kingdom, and the United States). Previous research in these countries indicates that White children, on average, demonstrate a preference for their racial ingroup by 3- to 5-years-old (Aboud, 2003; Aboud & Skerry, 1984; Best et al., 1975; Brand et al., 1974; Dunham et al., 2006; Kinzler & Spelke, 2011; Kircher & Furby, 1971; Lam et al., 2011; Perszyk et al., 2019; Weisman et al., 2015).

White children show a bias in favor of their ingroup across a wide range of racial outgroups, including favoring White people over Black people (Aboud, 2003; Best et al., 1975; Kinzler et al., 2009; Kinzler & Spelke, 2011; Kircher & Furby, 1971; Lam et al., 2011; Perszyk et al., 2019; Rutland et al., 2005), Latine people (Rice et al., 1974; Teplin, 1976), and Asian people (Dunham et al., 2006; Lam et al., 2011; Rutland et al., 2005). These ingroup biases also extend to resource allocation tasks, where White children have been shown to allocate more resources to an ingroup member over an outgroup member (Corbett et al., 2023; Mandalaywala et al., 2021; Renno & Shutts, 2015; Zinser et al., 1981). By the preschool years, White children demonstrate an understanding of race-based status hierarchies and apply them to judgments of who is “in charge” (Dukler & Liberman, 2022). Also, White children predict that a White child is more likely to live in a more expensive looking house or have higher-status belongings than a Black child (Mandalaywala et al., 2021; Olson et al., 2012; Shutts et al., 2016).

While White children tend to show fairly robust ingroup preferences across a wide range of measures and ages, children from minoritized racial groups show more variation across studies (Hailey & Olson, 2013). For example, Black children have demonstrated pro-Black preferences (Branch & Newcombe, 1986; Hraba & Grant, 1970; Newheiser & Olson, 2012), pro-White preferences (K. B. Clark & Clark, 1947; Jordan & Hernandez-Reif, 2009), and sometimes no difference in their evaluations or preferences for Black and White targets (Shutts et al., 2011). Fewer studies have examined Black children’s social judgments towards other racial groups (c.f., Black-Gutman & Hickson, 1996), at least within countries in which Black children are a numerical-minority group. Moreover, Black children’s race-based social judgments in countries where Black children are in the numerical majority group also yield mixed results. In two studies in South Africa, 6- to 12-year-old Black children (implicitly) preferred Coloured/Multiracial and White children over Black children (Dunham et al., 2014). Similarly, in rural Uganda, Black children preferred to play with a White child over a Black child (Marshall et al., 2022), and Black Brazilian children showed generally pro-White attitudinal preferences (Sacco et al., 2019). However, research in Cameroon found that Black preschoolers preferred to take a summer class taught by a Black adult, compared to a White or Chinese adult (Qian et al., 2016) and 6- to 12-year-old South African Black children showed no explicit preference for White over Black people and preferred Black people over Coloured/Multiracial people (Newheiser et al., 2014). Furthermore, sometimes no systematic biases have been observed. For example, Black South African 4- to 8-year-old children selected South African Black adults, White adults, Coloured/Multiracial adults, and foreign Black adults at equal rates when asked who they liked best (Shutts et al., 2011).

Much of the research on Asian children’s race-based social judgments has focused on Asian children in Asian countries where they are a majority group. The majority of this work, like the current study, has focused on East and Southeast Asian children, though some have also examined the judgments of South Asian children. This work has found that Chinese preschool-aged children prefer to be in a group with an Asian adult over a Black or White adult (Qian et al., 2016; Setoh et al., 2023), Japanese children show implicit and explicit preferences in favor of their own racial group over White and Black Americans (Dunham et al., 2006), Singaporean Chinese children prefer to befriend a child from their racial-ingroup (Lee & Setoh, 2022), and Malay and Chinese children demonstrate a preference for playing with White children over Black children (Steele et al., 2018). When exploring status, research in India demonstrates children ages 5- to 10-years-old were more likely to choose a White or light skin-toned South Asian child to be president of a fictional classroom (Santhanagopalan et al., 2022). Comparatively, less is known about the social judgments of Asian children living in countries in which they are the numerical minority. One study found that Asian (specifically Indian and Pakistani) elementary schoolers in the United Kingdom prefer to play with a same-sex Asian child compared to a same-sex Black or White child (Boulton & Smith, 1992). On the other hand, East Asian Canadian children ages 5- to 8-years-old rated pictures of White children as more social and competent compared to other East Asian and South Asian Canadian children (Friesen et al., 2012). This effect may extend into middle childhood, with additional work finding Chinese American children associate White children as having higher social status (Chen et al., 2019).

There is substantially less research with Latine children but what exists also presents mixed findings. Sometimes Latine children show a preference for White targets over Latine targets (Bernat & Balch, 1979; Dunham et al., 2007; Teplin, 1976) and other times Latine children show a preference for Latine targets over Black and White targets (Rice et al., 1974; A. D. Williams et al., 2023). Importantly, Latine children—particularly Mexican-American children—demonstrate a preference for dolls labeled as Mexican-American compared to those labeled White or Japanese, suggesting other ethnic-specific learned preferences (Kowalski, 2003).

### Challenges in Measuring Children’s Race Based Social Judgments

One challenge in comparing these results across studies and racial groups is that the actual tasks often vary considerably. Some ask children to choose between two adult targets they would want to be friends with (Kinzler & Spelke, 2011), others ask children how much they like a child target as indicated on a 4-point scale (Mandalaywala et al., 2021), and others ask children which of eight child targets they would rather sit next to in the classroom (Lam et al., 2011). Some studies utilize forced-choice measures (Kowalski, 2003) while others use child-friendly Likert-type scales (for a recent discussion of this methodological choice and its impact on results see deMayo & Olson, 2024). Most studies compare children’s attitudes across two racial groups, one of which is the child’s ingroup (Jordan & Hernandez-Reif, 2009; Marshall et al., 2022), though a few make a larger number of comparisons (e.g. Boulton & Smith, 1992). Similarly, some studies have directed children toward evaluating dolls (Hraba & Grant, 1970) whereas other studies use drawings (Kircher & Furby, 1971), cartoons (Jordan & Hernandez-Reif, 2009), or pictures of real people (Qian et al., 2016). Some focus on children as targets of evaluations (Dunham et al., 2014; Lee & Setoh, 2022; Marshall et al., 2022; Newheiser et al., 2014; Steele et al., 2018), while others focus on adults as targets of evaluations (Qian et al., 2016; Setoh et al., 2023; Shutts et al., 2011). Furthermore, measures of children’s understanding of racial differences in social status have varied in the aspect of status that they assess: wealth (Mandalaywala et al., 2020; Olson et al., 2012), social power (Dukler & Liberman, 2022; Heck et al., 2022; Mandalaywala et al., 2020; Santhanagopalan et al., 2022) and hierarchical status (Marshall et al., 2022; Yazdi et al., 2020). Thus, it has been difficult to determine whether differences across prior studies are due to the stimuli and type of task or due to differences in the participants themselves. The studies have also been conducted over the course of almost 80 years, which may be inadequate for assessing how children today consider race.

Adding to these interpretive challenges has been the wide variation in ages of children in these studies. This is a particular challenge because large-scale meta-analyses suggest that age is related to varying degrees of ingroup bias (Raabe & Beelmann, 2011), with children in the early elementary years generally exhibiting more racial bias compared to younger children (Aboud, 2003; Duckitt et al., 1999; Lee & Setoh, 2022). A meta-analysis exploring the emergence of racial prejudice found it develops and increases between early (2- to 4-years-old) and middle (5- to 7-years-old) childhood (Raabe & Beelmann, 2011). Several studies with White children have similarly suggested the emergence of ingroup preference by around 4- to 5-years-old (Aboud, 2003; A. Clark et al., 1980; Lam et al., 2011). Moreover, 3- to 4-year-old Singaporean children did not show an ingroup preference, but 5- to 6-year-old children did (Lee & Setoh, 2022). Thus, age and one’s cultural background may shift these judgments.

Past literature has examined the relation between different aspects of race-based social judgments. For example, several studies have suggested a link between racial attitudes (i.e., measures that assess good/bad judgments or liking evaluations of racial groups) and children’s beliefs about the social status of those racial groups (Marshall et al., 2022; Yazdi et al., 2020). Others have argued that there are good reasons to think that racial attitudes may not be linked to social status beliefs (e.g., we may like a group but not necessarily think of them as higher in status; Heck et al., 2022; Santhanagopalan et al., 2021). Similarly 3- to 5-year-old children’s race-based giving was not related to their racial attitudes (Renno & Shutts, 2015), suggesting racial attitudes and giving are not closely related. On the other hand, White adults with higher implicit racial bias against Black people chose to sit further away from a Black confederate (Amodio & Devine, 2006), suggesting there could be a link between people’s racial bias and interpersonal distance.

Lastly, a child’s social environment, (i.e., children’s contact with outgroup members and experiences with diverse others through school, neighborhood, or other interactions), also seems to influence their race-based social judgments. Previous research has demonstrated White children who attend racially White homogenous schools display negative attitudes towards racial minority child targets (McGlothlin & Killen, 2006) but that White children in racially heterogeneous schools may not (McGlothlin & Killen, 2010). Thus, a diverse social context may help reduce this effect. In further example, White children enrolled in White schools whose mothers reported having more racial minority friends showed lower levels of racial bias (Pahlke et al., 2012).

Schools are not the only aspects of a child’s social environment that seem to matter. For example, Singaporean Chinese children with other-race nannies have been shown to demonstrate less explicit racial bias towards that outgroup (Setoh et al., 2023). Additionally, Black, East and South East Asian, Latine, and White middle schoolers who made cross-ethnic friendships through extracurricular activities demonstrated more positive racial ethnic intergroup attitudes (Knifsend & Juvonen, 2017). Therefore, children who live in environments with varying exposure to outgroup members may have distinct race-based social judgments.

### The Present Study

In order to address some of these past difficulties in being able to directly compare children across racial groups and tasks, here we report on race-based social judgments from a large cross-sectional national study, the Childhood Intergroup Perceptions Study (ChIPS). This study investigated racial, gender, and novel group attitudes and related cognitions in East and South East Asian (henceforth referred to as Asian for clarity and brevity), Black, Latine, and White children distributed across five regions of the United States (Southern California, North Carolina, Connecticut, Hawaii, and Washington see further details below). This project has previously produced insight into gender attitudes (Halim, Atwood, et al., 2023) and gender socialization (Halim, Glazier, et al., 2023); this is the first report to focus on race-based social judgments.

In this project, each minoritized racial group was primarily recruited from two regions and White children were recruited in all five regions. All children completed a standardized task battery in a randomized order. Here we focus on the measures relating to race-based social judgments, namely race attitudes, race-related resource allocation, race-related social status judgments, and race-related social distance judgments (elicited via a hypothetical interpersonal distance task)^1^. The goal was to characterize the nature of early social judgments across these racial groups and tasks, as well as to explore relationships between tasks. We also explore links between these measures, percentage of outgroup members in participant’s county, and several individual difference measures including parents’ political orientation, socioeconomic status, and ingroup income at the county and national levels. Critically we do so using the same measures, scored the same way, in a diverse sample of children who are all in the same age range (4 to 6 years of age). This age range was selected because racial preferences in children are often found to broadly emerge around 4- and 5-years-old (Aboud, 2003; A. Clark et al., 1980; Lam et al., 2011).

## Methods

### Preregistrations and Connection to Larger Project

As part of the larger ChIPS study, and before beginning data collection, we created a study-wide methods preregistration that described the overall project’s recruitment procedures, stopping rules, and other details common across all of the anticipated subprojects making up the larger study. This study-wide methods preregistration along with modifications that were made as data collection proceeded are available at https://osf.io/jzwg3?view_only=33a431ac3305465ebacbfe7a623e2eb9. All modifications to the original plan were data-independent (i.e., we did not look at data before making decisions about modification). Modifications were based on recruitment challenges (e.g., COVID-19) that emerged over the course of data collection and are described below. Preregistrations, data, analyses, and supplemental information for the present study can be found at: https://osf.io/dsxm7/?view_only=cb456777ca7c45df80ebe8795c90d4ff.

Before data collection began, we separately preregistered distinct analysis plans for each of what we initially conceived of as distinct papers, each reporting on subsets of the data. However, because of redundancies across papers, extensive delays (e.g., COVID-19), and lack of funding for these projects after the grant ended, we decided to write one paper (this paper) to describe the overall findings on the child race-based social judgment measures, which also provides a stronger overall contribution to the field in being able to discuss findings across tasks. Providing this comprehensive overview of the racial judgment measures partially overlaps with three of the original preregistrations. For full transparency, analyses proposed in those preregistrations are included in the supplement, along with links to the original preregistrations on OSF. Other aspects of the larger project that focus on the child gender measures (Halim, Atwood, et al., 2023; Halim, Glazier, et al., 2023), the novel groups measure (Straka et al., under review), the face memory tasks (Pauker et al., in prep), and the parental socialization variables (Albuja et al, under review) have been published or are in the process of being published separately. In addition, we anticipate publishing the full dataset, linking all of these measures to one another, in a separate database publication for re-use by any research teams in the future.

### Preregistration and Modifications to Preregistration for Stopping Rule and Inclusion

In our initial methods preregistration we described a goal of recruiting 75 participants who were White from each of 5 U.S. research sites (Honolulu, HI; Seattle, WA; Long Beach, CA; New Haven, CT; Durham, NC). In addition, we recruited our three racial minority groups each at two sites, selecting these based on the largest minority groups at each site (Black participants in New Haven and Durham; Asian participants in Honolulu and Seattle; Latine participants in Long Beach and Seattle). We aimed to recruit 75 of each group at the two associated sites. In total, we therefore anticipated recruiting 375 White participants, 150 Black participants, 150 East or Southeast Asian participants (e.g., defined as of Chinese, Taiwanese, Mongolian, Japanese, Korean, Vietnamese, and Thai origin^2^), and 150 Latine participants (defined as of Mexican and/or Central American origin the original preregistration, later expanded via modification to include also South American or Caribbean). A child was considered to be in a racial group if both of their biological parents were identified in the parental survey as members of that racial group (though see below for one change to this criterion); if a child was adopted, we used whatever categorization parents provided for their child even if details about birth parents were not fully known to the adoptive parents. Within any group of 75 at a given site, we aimed for no more than 40 of one gender. In our preregistration, due to experimental constraints (e.g., providing same-race stimuli to participants) we specified that this was a project primarily about monoracial individuals (with the exception of White/Latine people, whom some see as multiracial, but whom we categorized as Latine given that some Latine people view Latine as an ethnicity and White—or another —as a racial categorization). Nonetheless we did enroll multiracial participants, and, as specified in our preregistration, report them in our supplementary analysis^3^. Following our preregistration we also excluded children with known developmental disabilities involving developmental delays or social processing (such as ASD) and children who did not speak fluent English (and therefore could not easily participate in the study), with the exception of Latine participants, for whom we had a Spanish version of the task. In total, 5 children were run in Spanish and 13 parents completed their questionnaires in Spanish. All other participants completed the study in English.

Unexpectedly, during the course of data collection, several events altered our ability to recruit as many participants as we had originally planned to recruit, and because this work was funded by grants, we needed to complete the project on time. Therefore we made several adjustments (in all cases, without looking at the data or the results when decisions were made). We increased the age range from 4–5 years to 4–6 years, aiming for at least 15 participants from each age (4, 5, 6) group and each racial group at each location, though many did not reach this goal (see below), and ultimately we had fewer 6 year olds than 4 and 5 year olds, due to the later addition of the 6 year olds combined with the earlier stopping than expected due to COVID-19. Also, seeing that many of the participants who were identified as Latine also identified as White (consistent with how the U.S. Census and NIH define Latine as an ethnicity rather than a race), we broadened our definition of Latine to include children who were described by parents as only Latine, and also included those described as Latine *and* White. We acknowledge that this category is potentially more heterogeneous than the others in this way.

Finally, we amended our stopping date twice. Initially, our goal was to end data collection in August of 2019, but some sites had struggled with recruitment so we extended the stopping date to August 31st, 2020. However, by March 2020, all labs were closed due to COVID-19, so in summer 2020, after it was apparent that in-person testing would not be permitted any time soon, we officially ended data collection. Most sites had not completed the expected data collection, but because the grant ended, COVID-19 continued, and one PI moved locations, it was deemed impossible to continue data collection and the team opted to publish the data available.

### Inclusion and Demographics of Participants in this Paper

Following the study-wide methods preregistration, we excluded participants from a specific analysis if they had not completed the tasks contributing to that analysis, but retained them for other analyses for which they did complete all tasks. Note that this entails different sample sizes for different analyses. In addition, we implemented preregistered exclusions for parental/teacher interference (e.g., if a parent/teacher entered the testing area or interfered with the task, *n* = 3). Full demographics for the final sample included in the current work are shown in Table 1.

Parents were given the opportunity to report their child’s ethnic background. Of those who chose to report, the top three ethnicities represented were Chinese, Japanese, and Vietnamese for Asian children, Mexican, Puerto Rican, and Peruvian for Latine children, and Italian, Irish, and English for White children. Of the Black families who reported their child’s ethnic background, the most frequently listed were “Black” or “African American.”

## Materials and Stimuli

The overarching study included several tasks that the present analyses do not report. These include gender-specific trials on each of the tasks below, a minimal groups measure, and a face memory task, all of which are or will be reported in other papers as detailed above. The order of measures and trials was counterbalanced to minimize the impact of measures on one another. In addition, parents reported some measures (primarily focusing on racial socialization behaviors) that are not explored in this paper but are the focus of another paper (Albuja et al., under review). Below we describe the stimuli and tasks relevant to the current paper.

Unique stimuli were used for each of the tasks such that children never saw the same picture twice. The status task used photographs of adults from The Chicago Face Database (Ma et al., 2015). All photos used were rated as clearly belonging to the perceived racial group (Asian, Black, Latine, White) based on Chicago Face Database ratings. Additionally, all photos were shown in color, and were adjusted to uniform size and resolution.

The photographs used in the resource allocation, attitudes, and interpersonal distance tasks were all selected from a large pool (*N* = 405) of photographs of children, including photographs from official data sets (e.g., The Cafe Dataset), photographs of children that were acquired through web searches, and photographs that the participating research labs had stored from various sources over the years. In order to select pictures for each of these tasks, we wanted to ensure that pictures were matched on age, affect, and attractiveness and that all pictures were generally seen by adults as members of the relevant race and gender groups. We had 10 adult raters (each of whom had spent considerable time working with children) independently estimate the approximate age of each child, 10 independent adult raters rate attractiveness (rated from 1 not attractive to 5 very attractive), and 12 adults rate affect (rated from 0 neutral to 4 happy). Ten adults independently categorized each photograph by race (options: Asian, Black, Latine, Multiracial, and White) and 10 adults independently categorized each photograph by perceived gender (writing in a comment if the child appeared to be gender nonconforming). Raters came from a range of racial and ethnic backgrounds. Only pictures in which there was > 90% agreement on gender identity and > 70% agreement on race were used. Interrater agreement was high for age (*SD* = 1.17, cronbach’s alpha = .92), attractiveness (*SD* =.78, cronbach’s alpha = .84), and affect (*SD* = .72, cronbach’s alpha = .96). We used these ratings to match stimuli across trials. All matches were within one standard deviation for age, attractiveness, and affect.

### Procedure

All participants completed the study in a quiet space (e.g., lab, school, or museum space), after their parents had provided consent. Participants who were monoracial Asian, Black, Latine (including Latine/White as described above), or White were run by an experimenter who had the same race as the participant, though not necessarily the same gender as the participant.

Before starting the tasks, the experimenter would obtain verbal assent. The experimenter also explained that participants could skip any questions they wanted to skip or stop the study at any time. After assenting, the experimenter would ask participants if it was okay to take a picture of them using a polaroid camera for an activity they would complete later. If participants agreed (98.9% of sample), the experimenter would take the participant’s picture using a polaroid camera and show children their picture. Otherwise a simple stick figure was used in place of a picture. The picture (or stick figure) was used in the Interpersonal Distance task described below.

Next, the experimenter explained that each participant would receive a ‘passport’ to track their completion of tasks in the study. After completing each task, participants got to stamp their passport. The six tasks were completed using Qualtrics on a tablet. Children completed these tasks in a randomized order, and it took approximately 30–40 minutes to complete the entire study.

Members of each research team piloted each measure and developed a formal protocol, then taught members at all other sites this protocol to ensure that procedures were standardized across sites. In addition, approximately once per month, each site would send a video of an experimenter running the protocol to a single site. Members of that team watched for any deviations from the protocol to ensure similarity in protocol throughout the testing period and across sites. This similarity across sites focused not only on wording and when/where the experimenter would point, but also standardized answers to common problems that arose (e.g., how to reply when a child pointed ambiguously or said “both” when that was not an option).

Either while the child was completing the study or in advance of the study (depending on location and availability of the parent), the parent or caregiver completed a questionnaire that is described below.

### Child Tasks

All tasks were completed in a randomized order. Trials within tasks were also randomized with two exceptions. Training trials always came before test trials and the Interpersonal Distance Task was blocked such that the order of the three blocks was set (though trials within blocks were randomized).

#### Attitudes Task

The goal of the attitudes task was to determine how much participants like members of various racial groups. Each target was rated independently (Dunham et al., 2011). On each trial, participants saw a face of a child and a 6-point face scale, which ranged from *really really don’t like* to *really really like.* Participants completed an initial training on the scale where they learned what each face meant (e.g., “So if you really really like something you can point to this face”). As part of the initial training, participants were asked what face they would point to if they really liked something, and what face to point at if they really disliked something. The experimenter confirmed that participants understood and pointed to the correct faces. Participants were then instructed to point to the face which best indicated how they felt about each target child (“how much do you like this kid? Can you point?”). Participants completed 16 race trials. Race trials included 4 gender-matched targets from each of the following groups: Asian, Black, Latine, and White.

#### Resource Allocation Task

The goal of the resource allocation task was to determine whether participants would differentially allocate resources to others based on the participant and target’s race (modified from Benenson et al., 2007 and Blake & Rand, 2010 to include multiple targets). Participants were told that they would see pairs of pictures of children, and that their task was to touch the image of the child to whom they wanted to give a colorful eraser. On each trial, the experimenter gave participants a new eraser to allocate.

Each trial included side-by-side pictures of two children. There were a total of 18 race trials. Pictures were matched to participant gender. There were three trials of each of the following combinations of children: Asian/Black, Asian/Latine, Asian/White, Black/Latine, Black/White, and Latine/White.

#### Interpersonal Distance Task

**Figure 1.**
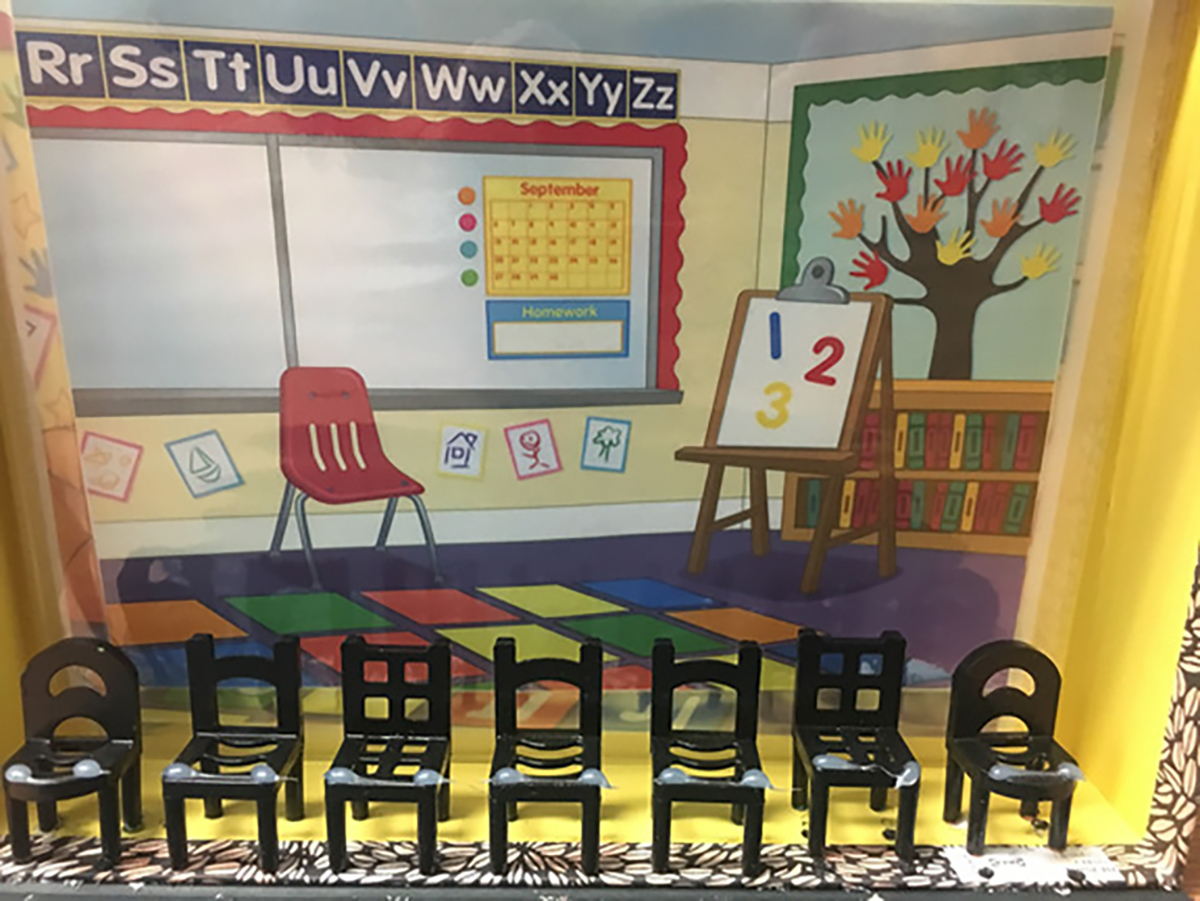
Photograph of the diorama presented to children in the interpersonal distance task

The goal of the interpersonal distance task was to determine whether participants preferred to be closer or further away from other children based on the other child’s race (adapted from Halim et al., 2017; also see Kawakami et al., 2007; Mehrabian, 1968; Word et al., 1974). The experimenter directed the participant’s attention to a diorama (See Figure 1) that had seven chairs lined up next to one another. The experimenter would then say, “Look! We are going to play a game where you imagine that you are going into a room, and you have to decide where to sit. See, we have these rows of chairs.” The experimenter would place a picture of a child on the far left or far right chair in the diorama, alternating the side across trials. Participants were given the polaroid picture of themselves to choose where they would want to sit. The experimenter recorded how far away from the target, the child placed their photograph, creating a scale from 1 to 6 seats away for each trial.

There were 3 blocks of 4 race trials for a total of 12 race-relevant trials. Each block had a different background (snack room, play room, classroom) and children were told they wanted to go to that room for a relevant reason (they were hungry, felt like playing, felt like learning). Each block included one picture of a same-gender child from each of the four race groups in this study. All race-relevant trials had pictures of children matched to participant gender.

#### Perceptions of Racial Groups’ Social Status Task

The goal of this status task was to determine whether participants associate race with social status (see Brey & Shutts, 2015). While distinct from direct measures of racial attitudes, past work has found that social status tends to predict the strength of intergroup attitudes (Bettencourt et al., 2001; Dunham et al., 2013), making it important to understand how it is perceived by children. To prompt children to think about something other than preferences, before this task, participants completed a filler task on which they saw a picture of a child eating broccoli for a snack and a picture of a child eating ice cream for a snack and were asked, “Which kid do you think likes vegetables the most?” After completing the filler task, participants moved on to the actual social status task, where they were asked to indicate social status by picking which adult was “in charge.”

To ensure that participants understood what “in charge” means, the experimenter defined being “in charge” as, “the person who makes all the rules, they’re like the boss.” Participants were then shown a series of paired photographs of adult faces and asked to point to who they thought was in-charge in each pair. There were 18 race trials where participants saw three trials of each pair: Asian-Black; Asian-Latine; Asian-White; Black-Latine; Black-White; Latine-White. All race trials were matched to participant gender. Trial order was randomized in Qualtrics.

### Caregiver Questionnaire

Caregivers completed a short questionnaire either on paper or online through Qualtrics that asked about demographics and the child’s socialization with regard to gender and race. Caregivers were told they could skip any questions they did not want to answer. Parents were asked their child’s sex (male, female, other\please specify); race (check all that apply) and were asked to specify ethnicity(ies) if known; child’s birthday; whether the child was born in the U.S. and if not, when they arrived in the U.S..

Caregivers were next asked a series of questions about themselves and (if applicable) a second caregiver. They were asked to indicate for each of these caregivers, whether they were a mom, dad, other relative, or “other”; whether they were an adoptive, biological, step, or other type of caregiver; their sex; highest level of education completed; employment status (full-time; part-time; other); country of origin; and racial and ethnic backgrounds (in addition, whether they identify as monoracial or biracial/multiracial). In addition, they were asked to provide their family’s estimated annual household income (in 5 divisions from under $25,000 to over $125,001); their political ideology (from 1-very liberal to 7-very conservative); languages spoken at home, and zip code. An overview of several of these demographic responses is provided in the supplement.

Caregivers were also asked to complete a scale about their child’s race socialization and gender socialization adapted from Hughes & Johnson (2001) that is not further discussed in this paper, but is the focus of other papers (race: Albuja et al., under review; gender: Halim, Atwood, et al., 2023). Lastly, To get a sense of how diverse the child’s immediate network is, parents were then asked to “Please write the initials of 5 children and 5 adults who spend the most time with your child.” They were asked to provide the race and gender of each of those individuals and for the children, to indicate if the individual was a friend or not, these network results are not further discussed in this paper, though we hope a future paper can focus on this topic.

## Results

### Overview of Analysis Strategy

For each of our four primary dependent measures we first present overall descriptive results collapsing across participant race. In order to examine potential differences by participant race we then report results of a linear regression predicting each dependent measure as a function of participant race, as well as independent tests of significance for each racial group considered separately^4^. To incorporate potential effects of age we then report an initial linear regression predicting each dependent measure as a function of (mean-centered) participant age, participant race, and their interaction. Given that our sample was somewhat sparser in the 6–7 age-range we also re-ran these models excluding children over the age of 6 to ensure that results reported in the main text still held; they did, so we focus our presentation on the full dataset. Following this, we present cross-task correlations as well as correlations between the four primary dependent measures and the several measures of participant and family environment described in the caregiver questionnaire, above. Note that due to planned exclusion criteria described above, and participants not always completing all measures, sample sizes differ somewhat for each measure. In all primary analyses, results are reported as ingroup preference relative to outgroup preference (with all outgroups combined), such that higher values indicate more ingroup preference (see Table 2 for calculation details).

### Ingroup Bias Overall and by Group

#### Attitudes Task

**Figure 2.**
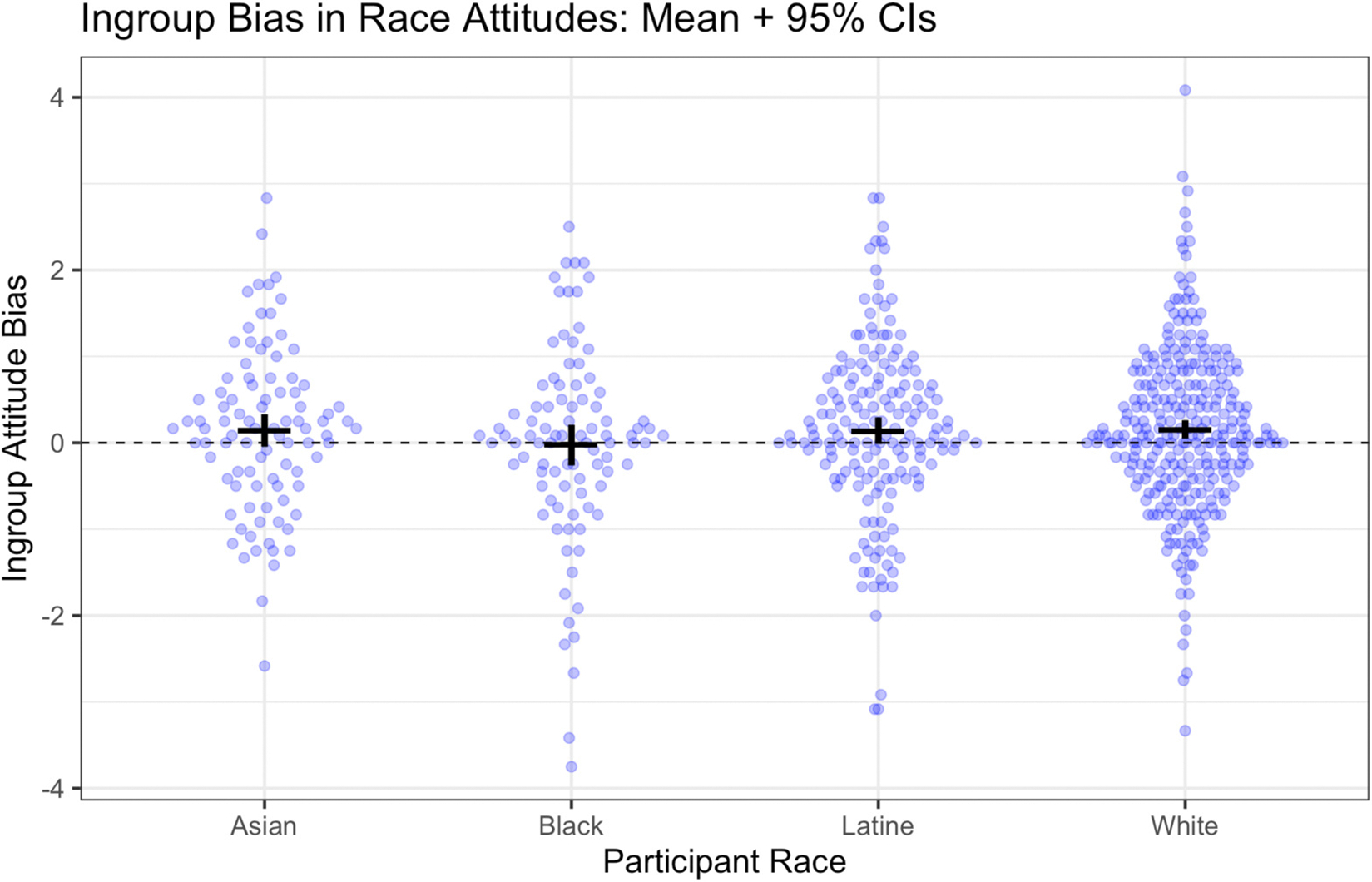
Ingroup bias on the attitudes measure, by participant race *Note.* Points reflect individual participants, jittered for ease of visualization. Horizontal black bars provide the mean; vertical black bars provide the 95% confidence interval around the mean.

As noted, our primary measure of racial attitudes is the difference score reflecting each participant’s average racial outgroup rating subtracted from the average racial ingroup rating. Summary statistics are presented in Table 2 and results are visualized in Figure 2. Overall, children showed ingroup race bias on the attitudes measure, though the effect was small by conventional standards, *M* = .12, *SD* = 1.00, *t*(642) = 3.13, *p* = .002, *d* = 0.12 \0.05; 0.20. Participant race did not predict the strength of attitudes, *F*(3, 639) = 0.75, *p* = .53. Somewhat complicating this interpretation, however, when race is considered separately, only White participants (the largest group) had an overall score that differed significantly from the zero-point of no bias, *M* = .15, *SD* = .95, *t*(288) = 2.67, *p* = .008, *d* = 0.16 \0.04; 0.27. Could these results reflect pro-majority, i.e. pro-White preference? This possibility is not supported by the data for several reasons. First, with the exception of Black participants, all groups showed a trend in the direction of ingroup preference (as visually displayed in Figure 2). Second, in a supplemental analysis examining the specific category contrasts in each trial, no racial/ethnic minority group showed a statistically significant preference for White children over their own group (see supplemental materials for full results). Across these analyses, gender did not appear as either a main effect or interaction with race (*p*s > .21).

#### Resource Allocation Task

**Figure 3.**
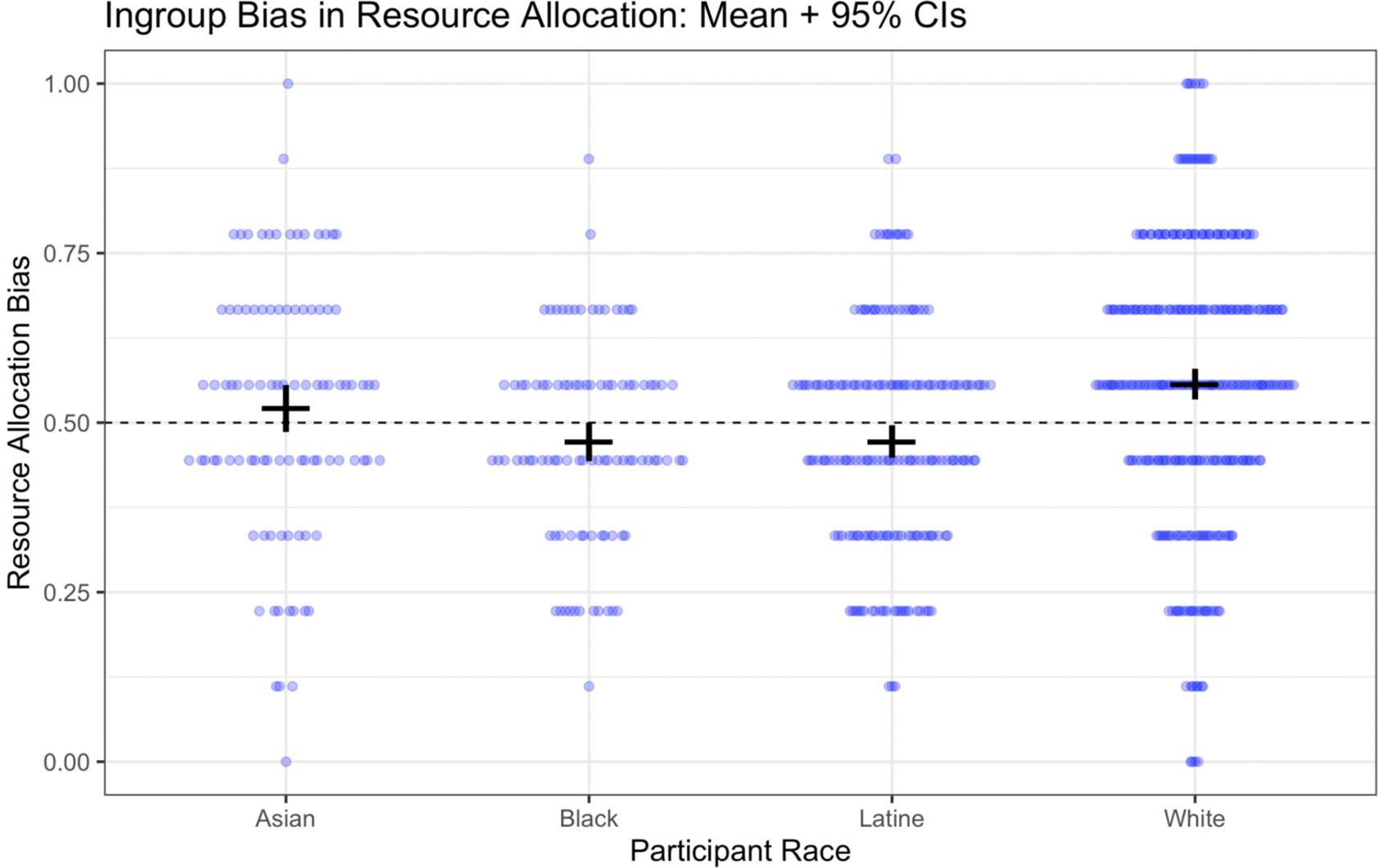
Ingroup bias on the resource allocation measure, by participant race *Note*. Points reflect individual participants. Horizontal black bars provide the mean; vertical black bars provide the 95% confidence interval around the mean.

Here, our primary measure was the deviation from chance responding, (i.e., the proportion of trials in which a participant chose to give the resources to their racial ingroup member minus .50 to index the deviation from chance). Results are visually displayed in Figure 3. Overall, participants displayed weak but statistically significant ingroup bias in their resource allocation behavior, *M* = .016, *SD* = .19, *t*(654) = 2.22, *p* = .027, *d* = 0.09 \0.01; 0.16. The effect of race as a predictor of resource allocation bias was significant, *F*(3, 651) = 9.85, *p* < .001. Tukey-adjusted pairwise comparisons indicate that White participants showed stronger ingroup favoritism on this measure than either Black or Latine children, both *p* < .001, but did not differ from Asian children, *p* = .37. No other pairwise comparisons reached statistical significance. Treated independently, the only group to show ingroup bias on this measure was White children, *M* = .06, *SD* = .20, *t*(291) = 4.72, *p* < .001, *d* = 0.28 \0.16; 0.39. Latine children actually tended to favor their racial outgroup, *M* = −.03, *SD* = .15, *t*(175) = −2.30, *p* = .023, *d* = −0.17–0.32; −0.02. Neither Asian nor Black children showed statistically significant bias on this measure. Gender did not moderate this effect either as a main effect or interaction with race (*p*s > .06).

#### Interpersonal Distance Task

**Figure 4.**
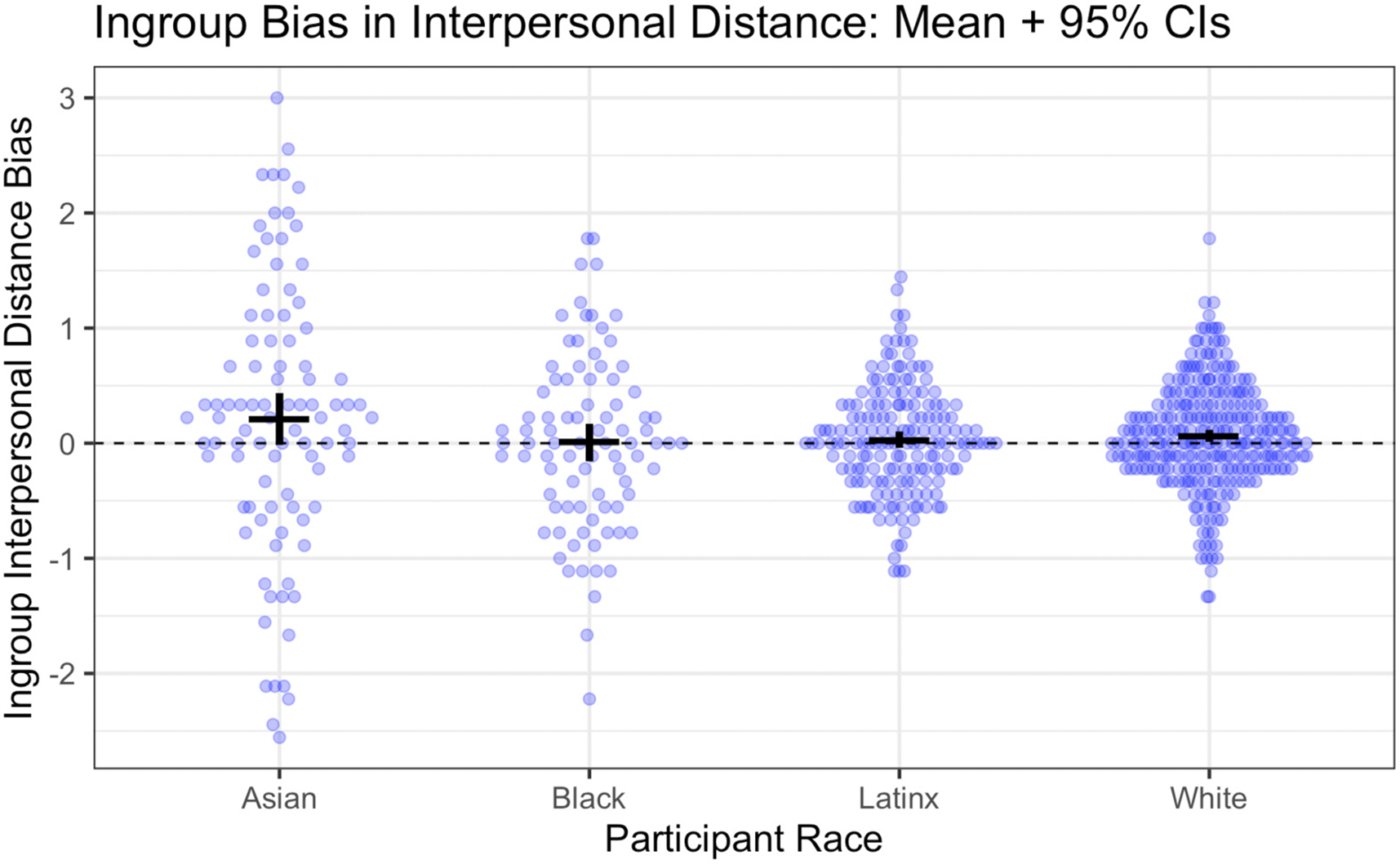
Ingroup bias on the interpersonal distance measure, by participant race *Note*. Points reflect individual participants, jittered for ease of visualization. Horizontal black bars provide the mean; vertical black bars provide the 95% confidence interval around the mean.

Here, our primary measure was the difference score reflecting the average distance between the participant and each outgroup target minus the average distance between the participant and each ingroup target. Summary statistics are presented in Table 2 and results are visualized in Figure 4. Overall, children showed ingroup race bias on the interpersonal distance measure, though the effect was small by conventional standards, *M* = .07, *SD* = .66, *t*(639) = 2.64, *p* = .009, *d* = 0.10 \0.03; 0.18. As with the attitude measure, this effect did not differ by participant race, *F*(3, 636) = 1.83, *p* = .14. However, again, only White participants had an overall score that differed significantly from the zero-point of no bias, *M* = .06, *SD* = .45, *t*(288) = 2.37, *p* = .019, *d* = 0.14 \0.02; 0.25. When incorporating gender into this analysis we observed a statistically significant interaction between gender and participant race, *F*(3, 632) = 2.93, *p* = .03. This effect was driven by the fact that bias on this measure was stronger in Asian girls than Asian boys but also stronger in Black boys than Black girls. However, we do not interpret this finding further because we did not predict gender effects, they were not consistently found across measures, and because neither the gender difference in Black or Asian children was independently significant.

#### Status Task

Here, our primary measure was the deviation from chance responding, (i.e., the proportion of trials in which a participant chose their racial ingroup member as the boss minus .50 to index the deviation from chance). Overall we saw no ingroup status bias on this measure, *M* = .00, *SD* = .18, *t*(644) = 0.28, *p* = .78, *d* = 0.01–0.07; 0.09. Analysis suggested that the effect of status did differ by race, *F*(3, 641) = 3.08, *p* = .027, but no Tukey-adjusted pairwise comparisons were significant. Considered separately, ingroup status bias was not statistically different from 0 in any participant racial group. Thus, there was no clear evidence of ingroup status bias in making judgments about social status among the sample. Gender did not moderate this effect either as a main effect or interaction with race (*p*s > .38).

Because status is so closely linked to the specific groups involved, in a supplemental analysis we also examined whether status judgments differed based on the particular comparison made by members of each racial or ethnic group. For example, did White participant status judgments differentiate White from Black, Black from Asian, and so on? This analysis, conducted separately for each participant group, revealed a statistically significant effect of comparison type for Asian participants, and White participants. It did not reach conventional levels of significance for Black or Latine participants. These results are presented in full in the supplement, but in brief, for Asian participants the only significant distinction was based on selecting more White than Asian participants as high in status (adjusted *p* = .003); for White participants, Asian targets were selected as high in status less often than both White (adjusted *p* = .007) and Latine (adjusted *p* = .049) targets. This suggests some children expect White adults to have higher status than other racial groups, but this expectation is not held consistently across participants groups or target groups.

### Effects of Age on Primary Dependent Measures

#### Attitudes Task

**Figure 5.**
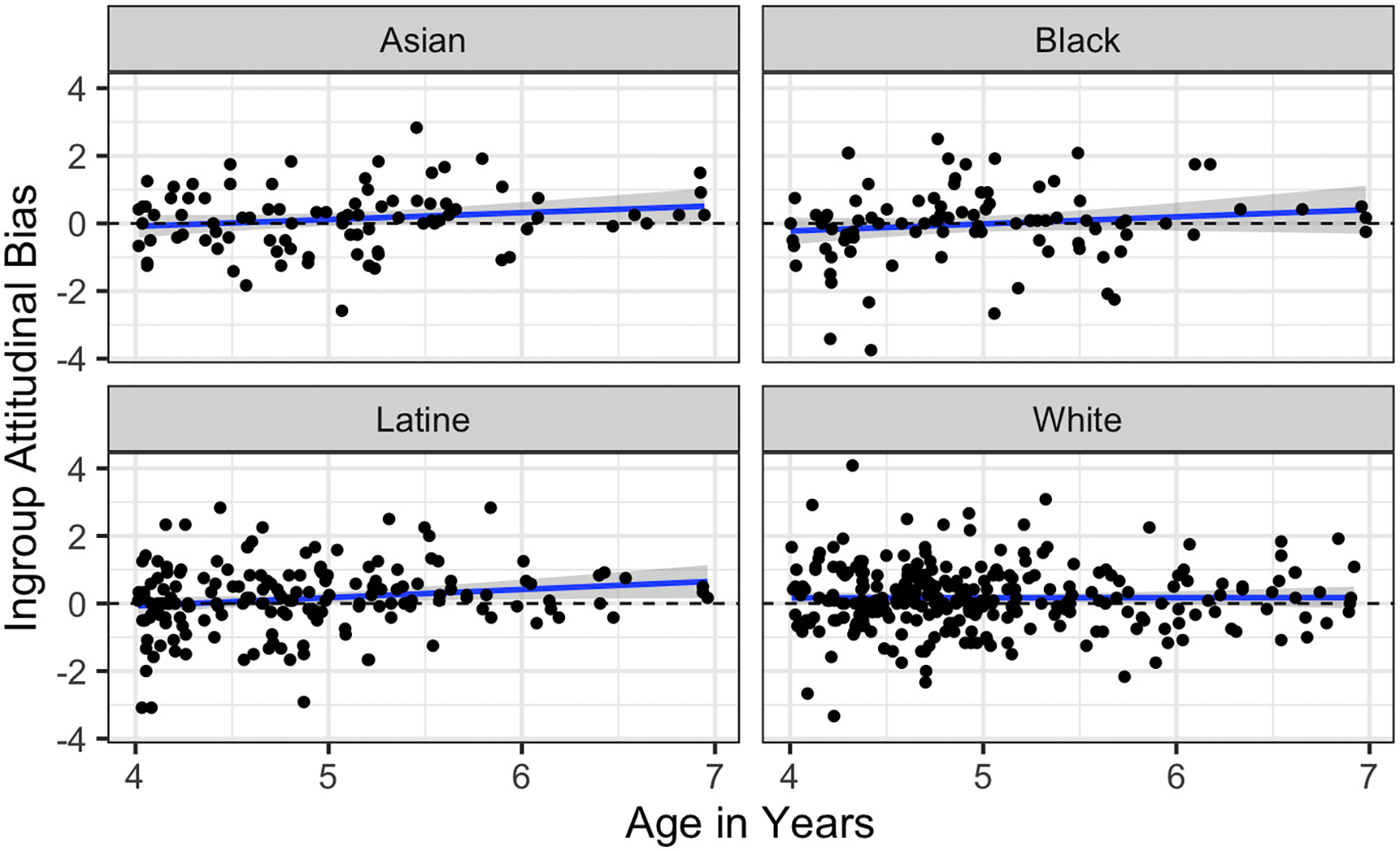
Effect of age on Attitudes Task

We next examined age effects by predicting ingroup bias via participant race and mean-centered participant age as well as their interaction. While we do not report it here, we also confirmed that all reported results for all measures hold when we restrict our sample to children under age 6, matching our original preregistration. The interaction term was not significant, *p* = .26, and so was dropped. In the resulting model the effect of age was significant and positive, *b* = .13, 95% CI .02; .23, *p* = .02, suggesting that the extent of ingroup bias tended to increase over this age range. These relationships are depicted in Figure 5.

#### Resource Allocation Task

**Figure 6.**
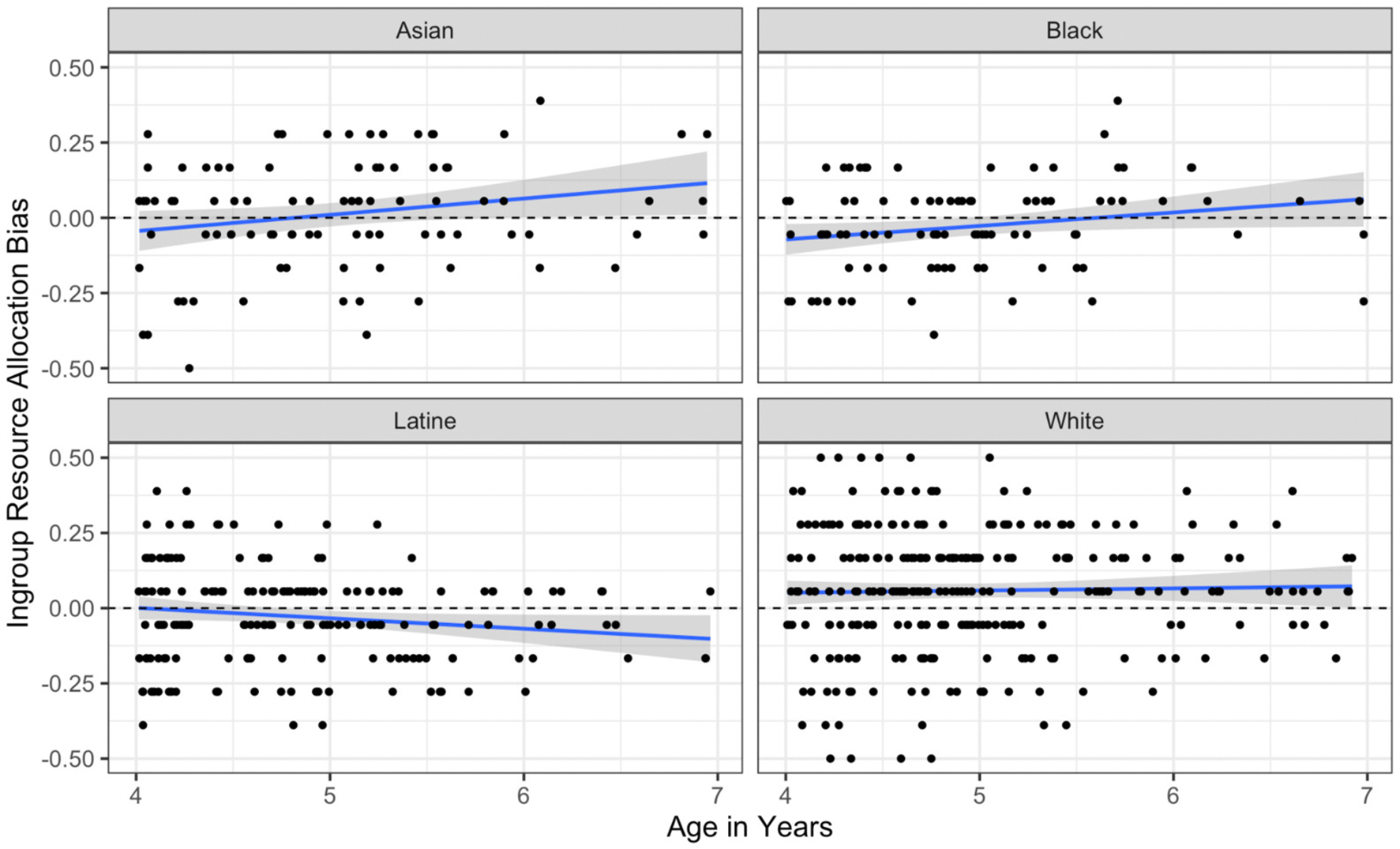
Effect of age on Resource Allocation Task

We next examined age effects by predicting ingroup bias via participant race and mean-centered participant age as well as their interaction for the resource allocation task. The interaction term was significant, *p* = .02, suggesting that the effect of age differed by participant race. We thus examined the simple slope of age for each racial group; see Figure 6. This analysis revealed statistically significant and positive effects of age on resource allocation bias in Asian, *b* = .05, 95% CI .00; .11, *p* = .04, and Black children, *b* = .28, 95% CI .06; .50, *p* = .01, but not in White children, *b* = .01, 95% CI −.03; .04, *p* = .68. The trend in Latine children was non-significantly negative, *b* = −.03, 95% CI −.07; .00, *p* = .053.

#### Interpersonal Distance Task

**Figure 7.**
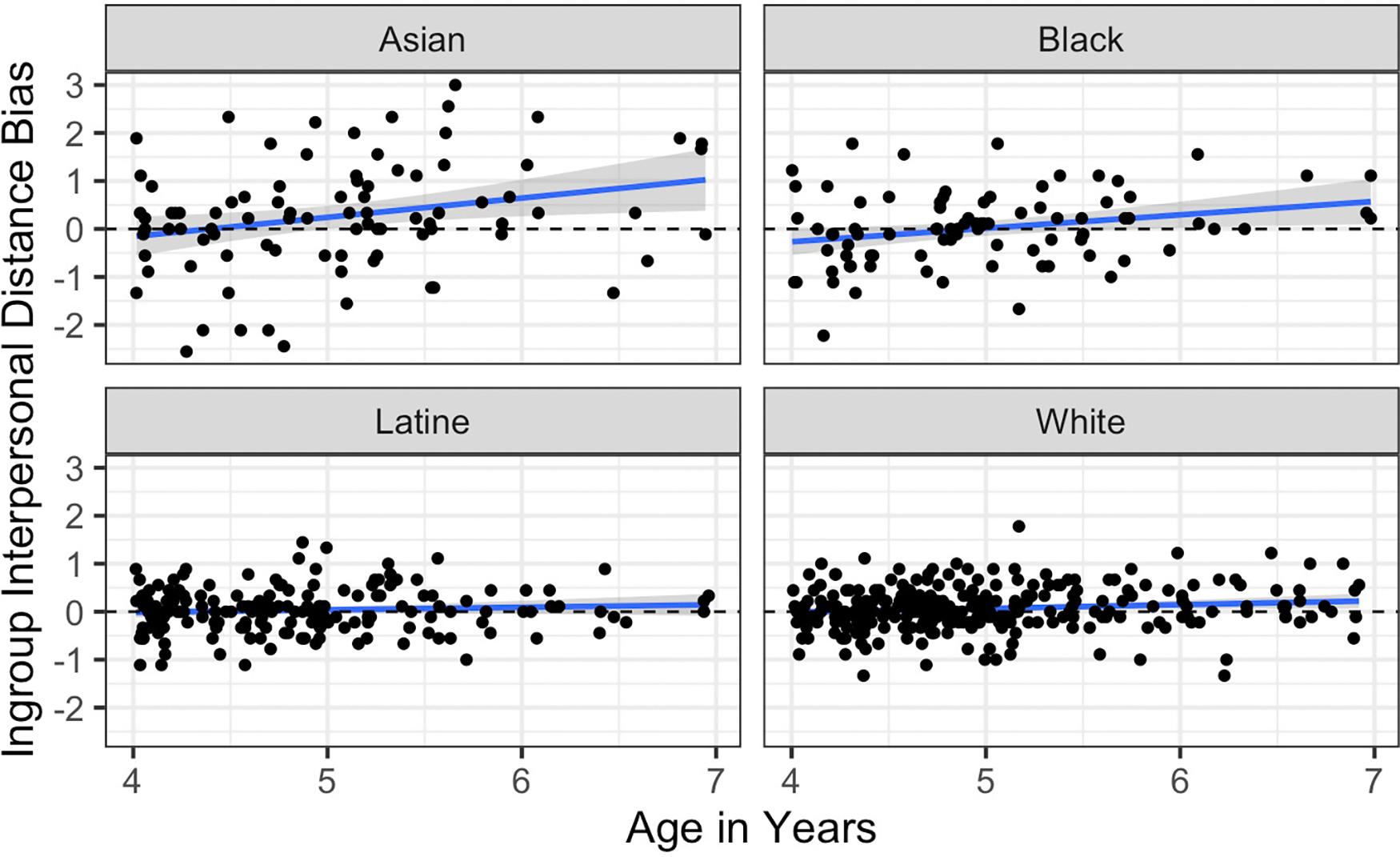
Effect of age on Interpersonal Distance Task

We again examined age effects by predicting ingroup bias via participant race and mean-centered participant age as well as their interaction. The interaction term was significant, *p* = .004, suggesting that the effect of age differed by participant race. We thus examined the simple slope of age for each racial group; see Figure 7. This analysis revealed that there was no statistically significant effect of age in Latine children, *b* = .05, 95% CI −.05; .16, *p* = .30. There was a small but significant effect of age in White children, *b* = .08, 95% CI .01; .15, *p* = .03, and somewhat stronger effects of age in Black children, *b* = .28, 95% CI .06; .50, *p* = .01, and Asian children, *b* = .40, 95% CI .08; .72, *p* = .01.

#### Status Task

We next examined age effects by predicting ingroup bias via participant race and mean-centered participant age as well as their interaction. The interaction term did not reach statistical significance, *p* = .07, and so was dropped. In the resulting model the effect of age was also not significant, *b* = 0.00, 95% CI −.02; .02, *p* = .98.

### Comparing White Children Across Five Regions

Given that White participants were included in all five regional samples, we conducted an analysis comparing race bias across our four main dependent measures in White children across the five sites (summarized in Table 3). Beginning with the attitudes measure, when predicting ingroup bias on the basis of data collection site the effect of site was not significant, *F*(4, 284) = .90, *p* = .46. However, inspection of the magnitude of bias in each site independently revealed that Hawaii appeared to be an outlier, with ingroup bias trending negative, *M* = −.22 (*SD* = .75), though non-significantly, possibly a consequence of this being our smallest sample of White participants (*N* = 23). Given the demographic differences between Hawaii and other research sites, namely that White children are a minority in Hawaii (Fojas et al., 2018), further examination of this potential difference could be an interesting future direction. There were no differences by site on the status measure, *p* = .97, resource allocation task, *F*(4, 284) = 1.53, *p* = .19, or interpersonal distance measure, *F*(4, 284) = 1.67, *p* = .16.

### Relationships Between Bias Measures

We also examined bivariate correlations between our four main dependent measures, (i.e., between ingroup-favoring attitudes, ingroup-favoring status judgments, ingroup-favoring resource allocation, and ingroup-favoring interpersonal distance decisions) (Table 4). However, these measures were largely unrelated (all *p* > .05) except for the relationship between ingroup-favoring status judgments and ingroup-favoring resource allocation. We note here that these were the two forced-choice measures and so could plausibly share more method variance. Given that and the small magnitude of the correlation we conclude that these measures were largely unrelated.

### Relationships with Parent Survey Measures, Environment, and Individual Difference Measures

Finally, we examined bivariate correlations between our four primary dependent measures and several measures drawn from the parent survey. In particular we focused on parental education and SES, parental political orientation, the racial composition of the child’s county (measures both as % outgroup and % White), and income ratios reflecting the relative income of the racial ingroup at both the county and national level. These correlations are presented in Table 5; given the large number of tests implied by this analysis we here interpret only correlations exceeding an uncorrected *p*-value of .01, though we display more conventional levels of significance in the table. On average correlations were relatively weak, though several statistically reliable correlations appeared between these variables and ingroup-favoring giving on the resource allocation task (Table 5). In particular, ingroup-favoring giving was more pronounced when the ingroup was relatively more wealthy than outgroups at both the county and national level, and ingroup-favoring giving was less pronounced when the county had a higher proportion of racial outgroup members.

## Discussion

The current study examined the prevalence of U.S. children’s race-based social judgments using standardized methods across five geographic regions. By testing 666 Black, Asian, Latine, and White 4- to 6-year-old children, we were able to provide a comprehensive evaluation of U.S. children’s racial attitudes, resource allocation, interpersonal distance, and status biases. Results indicated similar responses across three of the four measures: racial attitudes, interpersonal distance, and resource allocation tasks. Specifically, ingroup bias was observed on all three tasks when examined across all participants, regardless of one’s own racial background. Participants displayed more positive attitudes toward, preferred to sit closer to, and gave more resources to their racial ingroup, on average, consistent with a long history of research (Aboud, 2003; Aboud & Skerry, 1984; Best et al., 1975; Branch & Newcombe, 1986; Brand et al., 1974; Dunham et al., 2006; Hraba & Grant, 1970; Kinzler & Spelke, 2011; Kircher & Furby, 1971; Lam et al., 2011; Mandalaywala et al., 2021; Newheiser & Olson, 2012; Perszyk et al., 2019). When analyzed separately by racial group, White children showed significant ingroup biases on the attitudes, interpersonal distance, and resource allocation measures. In the resource allocation task specifically, White children showed a significantly stronger ingroup bias than Black or Latine children, but did not significantly differ from Asian children. Additionally, Latine children showed significant *out*group favoritism on the resource allocation measure, while Black and Asian children did not show a significant ingroup or outgroup effect. White children demonstrating significant ingroup bias while minority children are more variable in showing or not showing ingroup bias is consistent with previous work (Bernat & Balch, 1979; K. B. Clark & Clark, 1947; Dunham et al., 2007; Jordan & Hernandez-Reif, 2009; Teplin, 1976). Lastly, there were no significant racial group differences in the attitudes and interpersonal distance tasks.

The status measure was unique in that, when collapsing across all participants, results did not differ from chance; no ingroup or outgroup bias was observed. A more thorough discussion regarding the results of the four dependent variables are explored below.

### The Prevalence of Ingroup Bias in Attitudes, Interpersonal Distance, and Resource Allocation

Overall, White children in our sample tended to show significant ingroup bias across three of the measures, consistent with research suggesting that White children at these ages in the U.S. often show ingroup bias (Aboud, 2003; Aboud & Skerry, 1984; Best et al., 1975; Brand et al., 1974; Dunham et al., 2006; Kircher & Furby, 1971; Perszyk et al., 2019) and do so in comparison to a range of outgroups (Aboud, 2003; Best et al., 1975; Dunham et al., 2006, 2007; Kinzler et al., 2009; Kinzler & Spelke, 2011; Kircher & Furby, 1971; Lam et al., 2011; Perszyk et al., 2019; Rice et al., 1974; Rutland et al., 2005). However, although statistically significant, the effect sizes in this study were small or modest. Sometimes the White children showed ingroup bias that statistically differed from children in other racial groups (e.g., White children demonstrated significantly more ingroup bias than Black and Latine children, but not Asian children in the resource allocation task). However, on other tasks (e.g., attitudes and interpersonal distance tasks) there were no significant differences among racial groups. One methodological reason we might observe a more robust bias in White children when comparing them to other groups is that there were considerably more White participants in the sample, providing greater statistical power to observe a statistical difference from chance responding. Consistent with this interpretation, the means of the White group and other groups, and the effect sizes of the effects, were often similar in magnitude, suggesting that power may have been an issue here.

Another possible reason why White children may sometimes show more robust effects is that White people have relatively higher social, political, and material status in the U.S., as compared to all of the outgroups considered here (e.g., Black, Latine, and Asian children). In contrast, for all of the other groups, their outgroups include a mix of higher and lower status groups, or are all outgroups of higher status than their ingroup. If status is contributing to ingroup bias, as suggested by past research (Mandalaywala et al., 2021; Shutts et al., 2016), then we would expect these different degrees of ingroup bias in different groups as a function of their status position. We also might predict that the degree of ingroup bias depends on the specific outgroup comparison and whether that outgroup is higher status or not. We explored this question in brief in the supplement, where we provide summaries of each measure as a function of the comparison outgroup. However, those analyses do not provide clear support for this hypothesis.

We did not find evidence of variation in ingroup bias amongst White children in the five different geographic regions they were recruited from, despite the variation in the relative status and size of White people (in terms of being an overwhelming majority vs. minority) in each of those places. The one (albeit non-significant trend) that we judge to be worthy of specific future attention is a hint that White children in Hawaii may show *out*group bias on the racial attitudes measure. This is notable because Hawaii was the only geographic region tested where White people are a numerical minority and where there is a higher population of multiracial people. Additionally, previous research has demonstrated that children living in Hawaii have less essentialist views on race and exhibit less out-group stereotyping compared to children living in Massachusetts (Pauker et al., 2016, 2018). However, given that our sample of White children in Hawaii was also our smallest group (N = 23), and that this effect did not reach significance, we point to this as merely an important question for future research. Unfortunately, there was insufficient power to analyze differences in responses for Black, Asian, and Latine children across the regions they were drawn from.

### The Role of Age in Attitudes, Interpersonal Distance, and Resource Allocation

On the attitudes, resource allocation, and interpersonal distance measures, ingroup bias tended to increase with age. However, this age effect was observed to be significant for only a subset of racial groups on a subset of measures. For the attitudes measure there was a significant and positive effect of age for all groups. On the interpersonal distance measure, ingroup bias increased with age in White, Black, and Asian children but not in Latine children. Finally, on the resource allocation task, ingroup bias increased with age in Black and Asian children, but not in White and Latine children.

This finding of general increases in ingroup bias with age across this age range is consistent with meta-analyses about age-related racial bias across this age range (Raabe & Beelmann, 2011). Given that the pattern of which racial groups showed which effects is not consistent, our current interpretation is that there is generally a tendency toward an increase in bias but that estimates are less reliable if looking within smaller subsets of data.

This general finding aligns with other work to suggest that children’s understanding and use of race may be developing during this time frame. Work by Weisman and colleagues (2015), found that a sample of 3- to 6-year-old predominantly White children did not automatically encode the race of a target face despite showing racial preferences, but 8-year-olds did seem to encode race and demonstrate racial preferences. In contrast, for gender—another social category that is thought to be especially well-understood by children at these ages—3- to 6-year-olds showed both gender-based preferences and automatic encoding. Weisman etal.’s (2015) work, along with our own findings about age suggest that children’s understanding and use of race is continuing to develop during this developmental window, at least for White children in majority-White environments.

### Children’s Status Judgments

Children’s responses to the status measure were notably different from the other three measures in nearly all ways. The overall group mean did not differ from chance, indicating children did not show racial ingroup or outgroup bias on this measure. Since we observed preferences for children’s ingroup in the attitudes measure but no ingroup status judgment, our work provides evidence that children’s attitudes (i.e., how much they like a group) and social rank judgments can be dissociated. This finding is consistent with broader theorizing by Heck and colleagues’ (2022) who argue we should decouple our thinking about social attitudes and preferences from our thinking about social status. According to this view, these two social judgments need not necessarily track with one another, as one can like a group more and not see it as having higher rank (Santhanagopalan et al., 2021).

There was also no significant impact of age nor an interaction between age and race on this measure. Despite a significant effect of racial group, no individual comparison between groups reached statistical significance and no group’s mean differed from chance. One possible interpretation of these results is that children did not understand what “in charge” meant. However, we are skeptical of this interpretation because this method has been successfully used previously with children 3- to −10-years-old (Brey & Shutts, 2015; Dukler & Liberman, 2022; Heck et al., 2022). A related concern is that the phrase “in charge” may not be strictly capturing who children believe have the highest social status, which is what we’d intended to assess. Previous work has demonstrated that children will sometimes perceive a group as being “in charge” due to the group’s power (e.g., strength in numbers) and sometimes based on the group’s status (i.e., few people at the highest rank), depending on whether the group described was a numeric majority or minority (Heck et al., 2022). It is not clear how participants in our study interpreted it, or if that varied by child.

Our finding that children did not systematically tie race to social status differs from some past work that has found that children at these ages do associate race with status. Dukler and Liberman (2022) demonstrate that children indicate a White adult is “in charge” regardless of the race of the child participant. Indian children assign high status leadership positions to White children (their outgroup) and lighter-skinned South Asian children (a potential ingroup, although lighter skin is often associated with higher status within groups; Burton et al., 2010; Santhanagopalan et al., 2021). Chinese American children also assign higher status to White children (Chen et al., 2019), indicating children may not rely solely on ingroup status to decide who is higher status.

Past work that has found results suggesting children do link race to status has sometimes examined other aspects of status. For example, children seem to use race as a marker for status when the measure includes “wealth markers” such as higher-value belongings (Mandalaywala et al., 2020; Olson et al., 2012), but has not linked race to status when the status task implies social power (Mandalaywala et al., 2020). These findings and our own suggest that the type of status or other aspects of the task might impact whether children link race to status. Future research should consider assessing multiple aspects of power, such as the MacArthur ladder (Mandalaywala et al., 2020; Marshall et al., 2022), the “in charge” language, and wealth, to assess whether different measures and constructs produce different effects. Additionally, a more systematic analysis of social status tasks—including those known to be interpreted as indicative of distinct aspects of status (e.g., wealth, dominance, power, prestige)—and the links children do and do not make to race is needed.

Finally, it is possible race may not be seen by children as a reliable indicator of status in their communities; by contrast there is evidence that occupation, gender, and posture are status indicators at these ages (Dukler & Liberman, 2022; Halim, Atwood, et al., 2023; Legaspi et al., 2023). It is also notable that status is a less directly evaluative measure compared to our other three tasks, and in that sense it is perhaps not surprising that it patterned differently. However, given the robust links between status and ingroup attitudes (e.g., Bettencourt et al., 2001), we went into the project expecting similar patterns, as well as potential correlations between status and the other more directly evaluative tasks, something we did not generally observe.

### Relationships Between Measures

Despite similar effects on the attitudes, interpersonal distance, and resource allocation tasks, performance on those tasks were not related to one another. The only significant relationship was between ingroup status and ingroup giving in the resource allocation measure. Given the large number of correlations between tasks and the small size of that relationship, we remain cautious in interpreting the effect. In fact, we think the most likely explanation for the observed association is that these two tasks were the most similar to one another out of the four tasks—both tasks asked participants to make forced-choice decisions, suggesting that this association could indicate shared methods variance rather than a conceptual overlap between measures. Of course, at some point prejudicial attitudes do begin to predict prejudicial behavior (e.g., Kurdi et al., 2018), though even in adults only to a modest degree. Whether the lack of such relationships here is due to age or statistical power remains an open question.

### The Role of Demographic and Environmental Factors on Children’s Race-Based Judgments

We also explored the potential relationship between aspects of children’s environments and their responses on our racial social judgment tasks. We generally found no or limited evidence for clear relations between these measures. One exception was for the resource allocation task. We found that children who lived in counties in which their ingroup had higher incomes, and children whose racial ingroups had higher incomes in the U.S., tended to give more resources to their ingroup. We also found that children in counties with higher percentages of outgroup members (i.e., children in more racially diverse counties) tended to show less ingroup bias on this resource allocation measure. This is consistent with previous research that demonstrates exposure to people from other racial groups can decrease levels of ingroup bias (Bernat & Balch, 1981; Pahlke et al., 2012; Setoh et al., 2023). However, it is important to note that none of these effects were very large (*r* ~.10-.15), so these factors appeared to play a relatively small role in predicting resource allocation behavior.

### Limitations and Future Directions

While this study had strengths, including its relatively large and diverse sample, diverse stimuli tailored to participants’ own identities, and the systematicity with which we assessed race-based social judgments, there were also limitations. In part due to disruptions relating to the COVID-19 pandemic, we did not ultimately recruit our full proposed and pre-registered sample, giving us less statistical power than we had initially envisioned, especially for racial minority participants. We also did not have participants from each racial group in every geographic location, which would have been helpful to better disentangle potential race and geographic differences. Thus, we only reported geographic analyses for our White sample. Additionally, despite the fact that our stimuli were pre-tested and rated as part of their respective group (e.g., Asian, Black, Latine, White by 70% or more of the raters), it is possible that the stimuli’s skin-tone did not match (at least some) participants’ own skin-tone across all groups. This potential mismatch may have influenced participants’ responses throughout the study. This is because there is a known positive bias towards people of lighter skin even within racial groups (i.e., colorism; Burton et al., 2010). Colorism has previously been observed in Latine (Kaufman & Wiese, 2012), Asian (Gao et al., 2024; Santhanagopalan et al., 2022), and Black children (T. L. Williams & Davidson, 2009). Thus, our Latine, Asian, and Black samples whose skin-tone did not match the stimuli could have had differing perceptions of who belongs to their ingroup vs outgroup and preference towards lighter-skinned stimuli could have affected individual evaluations. Future work should address these critical skin tone variations in existing and new stimuli selection.

An additional limitation is that groups such as Asian, Latine, Black, and White are not monoliths and may have substantial within-group variation that we are unable to capture in the current study. This is because there was unfortunately not enough statistical power to assess differences between the different Asian, Latine, Black, and White ethnic groups that were reported by parents. Future research should investigate ingroup preferences within racial groups (e.g., are there differences between Chinese and Japanese children’s race-based social judgements?), which could give insight into who different groups consider to be ingroup vs. outgroup members.

Our studies were also limited by the particular instantiation of each measure that we selected. Our attitudes measure, for example, was an absolute measure, while our resource allocation measure was a forced-choice measure. Recent work suggests that within a single domain, the effect size of social group bias (e.g., the degree of ingroup race preference) varies such that absolute measures tend to produce smaller effect sizes than relative measures (deMayo & Olson, 2024). This may explain why the ingroup effect, particularly for White children on the preference attitudes, is smaller in this study than in other studies (Perszyk et al., 2019; Qian et al., 2016).

Overall, this study presented an opportunity to pull together common methods, administer them to diverse samples of children in five distinct geographic locations, and assess potential generalizability. Adopting this approach allowed us to confirm that White children show the most consistent ingroup bias, while racial minority children’s ingroup bias levels remain more variable. This variability could be due to the internalization of prevailing status norms that privilege White people—a phenomenon that relates to reported findings in South Africa and Uganda, where even though White people are not the numeric majority, they still hold significant power and capital, and where Black African children show preferences for White people (Dunham et al., 2014; Marshall et al., 2022; Newheiser et al., 2014). Lastly, we further hope that the approach adopted here—a multi-site test with shared methods, continuous monitoring during the course of data collection to confirm similarity in procedures across sites, and a racially and ethnically diverse sample—is adopted more widely.

## Supplementary Material

1

## Figures and Tables

**Table 1.** Full demographics for the final sample based on parent report

		Asian (N = 94)	Black (N = 95)	Latine (N = 179)	White (N = 298)

**Age** *M(SD)*		5.05 (0.75)	4.96 (0.70)	4.84 (0.69)	4.97 (0.70)

**Gender**	Female	47	43	80	144
	Male	47	52	99	154

**Site**	Durham, NC	1	59	9	83
	Honolulu, HI	39	0	1	23
	Long Beach, CA	0	0	85	35
	New Haven, CT	0	36	2	70
	Seattle, WA	54	0	82	87

**Table 2.** Means, standard deviations, Bayes Factors (BFs), and t-test results for each participant racial group and for the entire sample on the four primary dependent measures.

							95% CI for Cohen’s d		
	
		M(SD)	t	df	p	BF	Cohen’s d	Lower	Upper

**Asian** (N = 94)	**Attitudes**	0.15 (0.94)	1.53	92	.13	.78	0.16	−0.05	0.36
	**Resource Allocation**	0.021 (0.19)	1.05	92	.30	.48	0.11	−0.10	0.31
	**Interpersonal Distance**	0.21 (1.20)	1.70	91	.09 ~	.97	0.18	−0.03	0.38
	**Status**	−0.035 (0.18)	−1.82	90	.07 ~	1.2	−0.19	−0.40	0.02

**Black** (N = 95)	**Attitudes**	−0.021 (1.10)	−0.18	91	.86	.32*	−0.02	−0.22	0.19
	**Resource Allocation**	−0.028 (0.15)	−1.86	93	.07 ~	1.2	−0.19	−0.39	0.01
	**Interpersonal Distance**	0.014 (0.76)	0.17	85	.86	.32*	0.02	−0.19	0.23
	**Status**	−0.028 (0.18)	−1.54	93	.13	.79	−0.16	−0.36	0.04

**Latine** (N = 179)	**Attitudes**	0.14 (1.0)	1.79	168	.08 ~	.95	0.14	−0.01	0.29
	**Resource Allocation** ^	−0.028 (0.16)	−2.30	175	.023*	2.3	−0.17	−0.32	−0.02
	**Interpersonal Distance**	0.03 (0.47)	0.83	172	.41	.32*	0.06	−0.09	0.21
	**Status**	0.012 (0.17)	0.93	167	.36	.35	0.07	−0.08	0.22

**White** (N = 298)	**Attitudes** +	0.15 (0.95)	2.67	288	.008**	4.8*	0.16	0.04	0.27
	**Resource Allocation** +	0.06 (0.20)	4.72	291	< .001**	4086**	0.28	0.16	0.39
	**Interpersonal Distance** +	0.063 (0.45)	2.37	288	.02*	2.4	0.14	0.02	0.25
	**Status**	0.018 (0.19)	1.61	291	.11	.62	0.09	−0.02	0.21

**Total** (N = 666)	**Attitudes** +	0.12 (1.0)	3.13	642	.002**	13.6**	0.12	0.05	0.20
	**Resource Allocation** +	0.016 (0.19)	2.22	654	.027*	1.4	0.09	0.01	0.16
	**Interpersonal Distance** +	0.069 (0.66)	2.64	639	.009**	3.6*	0.10	0.03	0.18
	**Status**	0.002 (0.18)	0.28	644	.780	.14*	0.01	−0.07	0.09

Note.

** *p* < .01 or BF ratio > 8

* *p* < .05 or BF ratio > 3

~ *p* < .10.

All reported scores are difference scores comparing ingroup responses to outgroup responses, with positive values indicating a relative preference for one’s ingroup (average racial ingroup rating - average racial outgroup rating)

+ indicating a significant preference towards one’s ingroup and

^ indicating a significant preference towards one’s outgroup.

BFs reflect the relative strength of the evidence for ingroup bias as compared to a lack of ingroup bias on the stated measure, computed with a Cauchy prior scaled such that 80% of effects are between −.7 and .7; a value > 1 indicates relatively stronger evidence in favor of the presence of ingroup bias while a value < 1 indicates relatively stronger evidence in favor of the absence of bias.

**Table 3.** Comparing White participants’ responses on all four measures across all five sites

	Attitudes *M(SD)*	Resource Allocation *M(SD)*	Status *M(SD)*	Interpersonal Distance *M(SD)*

**Durham, NC**	.18 (.85)	.07 (.20)	.01 (.19)	.04 (.41)
**Honolulu, HI**	−0.22 (.75)	.10 (.16)	.03 (.20)	−.08 (.63)
**Long Beach, CA**	.16 (1.15)	.08 (.23)	.03 (.21)	−.04 (.46)
**New Haven, CT**	.20 (1.0)	.01 (.22)	.02 (.18)	.15 (.50)
**Seattle, WA**	.18 (.96)	.07 (.19)	.01 (.18)	.09 (.39)

**Table 4.** Correlations between four main dependent measures.

	Attitudes	Status	Resource Allocation

**Attitudes**	-	-	-
**Status**	−0.02	-	-
**Resource Allocation**	−0.02	0.09*	-
**Interpersonal Distance**	0.03	0.04	0.00

* p < .05

**Table 5.** Correlations between parental and environment measures and four main dependent measures of racial bias

	Attitudes	Resource Allocation	Interpersonal Distance	Status

**Parent political orientation**	.10*	−.02	−.04	.02
**Parent socioeconomic status**	.03	.08	.03	.03
**Pct Outgroup members in county**	−.06	−.14***	−.00	−.01
**Pct White in the county**	.02	.01	.07	.01
**Relative ingroup income, national level**	.03	.13**	.10*	−.02
**Relative ingroup income, county level**	.06	.14***	.05	.01

Note.

* (uncorrected) *p* < .05

** (uncorrected) *p* < .01

*** (uncorrected) *p* < .001.

## Data Availability

All the stimuli, presentation materials, participant data, and analysis scripts can be found on this paper’s project page: https://osf.io/dsxm7/?view_only=cb456777ca7c45df80ebe8795c90d4ff

## Footnotes

1 The only race related measure we do not include in this paper that was part of the battery of measures for children was a measure of race memory” should be edited to read “The only race related measure not included in this paper was a battery of measures on children’s race memory. We do not include that measure because it is the focus of a different planned paper and was conceptually different from the present measures, in that it is not a clear measure of racial attitudes or biases.

2 We specified these ethnic subgroups because photo stimuli were matched with participants’ race, and some subgroups were not represented in our stimuli (e.g., stimuli did not include people with South Asian appearance, so we specified the category with a focus on East and South East Asian participants).

3 One key challenge for interpretation of the multiracial data is that the study design includes “own race” stimuli. However, we did not have multi-racial stimuli and therefore they saw monoracial stimuli selected to match one of the races making up the child’s racial background. Similarly, they were run by a monoracial experimenter from one of the racial backgrounds in the child’s background. Due to this constraint in study design, interpretation of “in” and “out” groups was complicated and different for multiracial children than for other children. Further, within the multiracial group, interpretations of data are challenging. For example, a Black-White biracial child might have seen Black and White stimuli, two ingroups, but a Black-Asian biracial child might have seen Black and White stimuli, therefore representing an in and an outgroup. For these reasons the results from multiracial participants should be interpreted with caution and are presented in the supplementary materials only.

4 Geographic diversity was not included as a random effect in the models because location is confounded with participant race due to the fact that each racial group other than White was specifically recruited at only two sites.
