# Supplementary material for: Children’s Use of Race in Their Social Judgments: A Multi-Site, Multi-Racial Group Comparison
Source: Collabra Psychol. Author manuscript; Available in PMC 2025 Dec 17. (PMC12707373; doi:10.1525/collabra.132489)
Supplement: 1 [file NIHMS2111994-supplement-1.pdf]

Supplementary Materials

Peer Review Communication

Download: https://collabra.scholasticahq.com/article/132489-children-s-use-of-race-in-their-social-judgments-a-multi-site-multi-racial-group-comparison/attachment/272132.docx?auth_token=pFmzIIVjmL71LFwLAkDc

---

Response to Reviewers

Download: https://collabra.scholasticahq.com/article/132489-children-s-use-of-race-in-their-social-judgments-a-multi-site-multi-racial-group-comparison/attachment/272133.pdf?auth_token=pFmzIIVjmL71LFwLAkDc

---

Response Letter

Download: https://collabra.scholasticahq.com/article/132489-children-s-use-of-race-in-their-social-judgments-a-multi-site-multi-racial-group-comparison/attachment/272134.pdf?auth_token=pFmzIIVjmL71LFwLAkDc

---

Supplemental Material

Download: https://collabra.scholasticahq.com/article/132489-children-s-use-of-race-in-their-social-judgments-a-multi-site-multi-racial-group-comparison/attachment/272135.pdf?auth_token=pFmzIIVjmL71LFwLAkDc

---

Downloaded from http://online.ucpress.edu/collabra/article-pdf/11/1/132489/871050/collabra_2025_11_1_132489.pdf by guest on 15 September 2025
